# Evaluating Stabilizers Effects on Aroma Release Dynamics in Ice Cream: A PTR‐ToF‐MS Analysis

**DOI:** 10.1002/jms.70092

**Published:** 2026-07-30

**Authors:** Camila Cossettin Teixeira, Michele Pedrotti, Lorenzo Gennari, Andrea Cavallero, Flavia Gasperi, Iuliia Khomenko, Franco Biasioli

**Affiliations:** ^1^ Fondazione Edmund Mach San Michele all'Adige Italy; ^2^ Center Agriculture Food Environment C3A University of Trento San Michele all'Adige Italy; ^3^ Soremartec Ferrero Group Alba Italy

**Keywords:** aroma release, ice cream, peach, PTR‐ToF‐MS, stabilizer

## Abstract

Capturing the dynamic volatilome of frozen matrices during phase transitions remains an analytical challenge, typically requiring invasive sampling. This study employs proton transfer reaction time‐of‐flight mass spectrometry (PTR‐ToF‐MS) as a rapid, noninvasive technique that directly samples headspace volatiles without preconcentration to efficiently monitor four peach‐related aroma compounds (isoamyl acetate, 3‐hexenyl acetate, linalool, and furaneol) from frozen to melted ice cream. The samples vary in stabilizer composition, including a control (no stabilizer), single‐gum, binary, and ternary blends. Monitoring occurred in the frozen state (incubation for 1 min at 4°C) and after melting (incubation for 20 min at 30°C). Results demonstrate that all stabilizer blends attenuated volatile release (average reduction in total aroma release of 40.39%) compared with the control, although statistically significant formulation effects were compound dependent and were not observed for furaneol. The magnitude of suppression was dependent on both stabilizer composition and the physicochemical properties of the aroma compounds. Notably, individual aroma compounds displayed differential release patterns across formulations; isoamyl acetate generally showed a more pronounced release than cis‐3‐hexenyl acetate in specific stabilized formulations (LGK and LAK). Stabilizers that consistently suppressed peach‐related volatiles also demonstrated reduced total odor activity values. These results validate PTR‐ToF‐MS as an effective tool for monitoring the volatilome of frozen matrices and tracking aroma release during melting, illustrating how stabilizer selection can be optimized for aroma delivery in ice cream.

## 1 Introduction

Ice cream is a complex multiphase colloidal system composed of ice crystals, air bubbles, fat droplets, and a concentrated unfrozen aqueous phase [1]. This intricate microstructure influences flavor delivery through physicochemical interactions among phases and dynamic changes during oral consumption [2, 3]. As ice cream is eaten, it undergoes a phase transition from semisolid to liquid due to warming and dilution with saliva, which alters the partitioning and release of volatile compounds (VOCs) [4].

Ice cream flavor and aroma release are primarily driven by fat content and its solid‐to‐liquid profile, with additional modulation arising from protein‐aroma interactions. Although aroma perception evolves throughout consumption as the food matrix warms and mixes with saliva, early‐stage volatile release has been shown to be strongly associated with overall aroma delivery [5]. Aroma release is a key driver of sensory perception and consumer acceptance, as the intensity and timing of aroma delivery strongly shape the consumption experience [5, 6]. Consequently, understanding how structural features of ice cream, including the presence of stabilizers, modulate aroma release is critical for optimizing flavor and overall product quality. However, despite the well‐established role of stabilizers in controlling texture attributes and melting resistance, their specific influence on aroma release dynamics during frozen‐to‐melted transitions remains insufficiently characterized.

Stabilizers increase mix viscosity by binding to water, thereby limiting crystal growth and delaying the occurrence of graininess during storage. When used in blends, stabilizers offer synergistic effects, leading to smoother texture, improved meltdown resistance, and overall product stability throughout shelf life [7, 8]. Among the most commonly used stabilizers are galactomannans, such as locust bean gum (L) and guar gum (G), which have a β‐(1 → 4)‐linked mannose backbone. These polysaccharides differ in their degree of galactose substitution, expressed as the mannose‐to‐galactose (M/G) ratio [9]. A lower M/G ratio, as found in guar gum, promotes greater polymer–water interactions and hydration, whereas a higher M/G ratio, characteristic of locust bean gum, favors polymer‐polymer associations and network formation. Consequently, the M/G ratio influences the balance between thickening and structuring effects, affecting water mobility, ice crystal stability, and the resistance of ice cream to structural collapse during melting [10].

Beyond their texturizing properties, hydrocolloids significantly influence VOCs diffusion and partition among the phases (fat‐air‐unfrozen serum) [11, 12]. Understanding the mechanisms by which stabilizers modify aroma release is therefore critical for designing formulations that maximize aroma perception without compromising texture. Previous research has shown that stabilizers can affect volatile release in two principal ways: (i) by increasing the viscosity of the matrix, which slows aroma compound diffusion, and (ii) by forming molecular interactions such as hydrogen bonding, encapsulation, complexation, or adsorption that selectively bind or entrap aroma molecules [13, 14]. The net effect varies widely with the type and concentration of the stabilizer, as well as with the physicochemical properties of the volatiles themselves, particularly their molecular weight, polarity, and hydrophobicity [15].

Moreover, temperature‐dependent factors become especially relevant in ice cream, where freezing, melting, and phase transitions during consumption further complicate volatile behavior. A higher fraction of ice crystals reduces aroma mobility, while partial melting in the mouth rapidly increases the rate at which VOCs diffuse into the headspace [16]. Studies on stabilizer effects in dairy‐based systems consistently highlight the dual importance of both viscosity and molecular interactions in shaping aroma release [14, 17]. For instance, small variations in guar gum concentration (on the order of 0.02% to 0.8% w/w) can significantly alter diffusion rates [14], while high sugar or polysaccharide contents (e.g., sucrose above 20%–25% w/w), strongly suppress or delay volatile release, due to thermodynamic partitioning and compound–matrix interaction [18]. Additionally, thermodynamic and kinetic analyses underscore how compound‐specific binding affinities, fat crystallization, and interfacial behaviors modulate aroma release [19, 20].

Predominantly, headspace solid‐phase microextraction coupled with gas chromatography mass spectrometry (HS‐SPME‐GC‐MS) and related GC‐MS methods have been applied to ice cream matrices, varying in stabilizer type, fat level, and source, and often protein content [21, 22, 23, 24]. Studies manipulating fat levels and sources (e.g., milk fat vs. vegetable fat) have demonstrated that low‐fat formulations release higher headspace concentrations of hydrophobic volatiles compared with high‐fat analogs and that fat replacers, such as whey protein or maltodextrin, further alter volatile profiles by modifying the matrix microstructure [5]. Investigations of stabilizer blends have shown that stronger hydrocolloid networks retard the release of small and highly VOCs, with HS‐SPME‐GC‐MS revealing distinct headspace profiles for each stabilizer type [25]. While GC‐MS remains the gold standard for the qualitative and quantitative analysis of food volatiles, its inherent time requirements and sample preparation steps make it less suitable for high‐throughput screening or real‐time monitoring of frozen matrices.

In this context, alternative approaches are increasingly necessary, featuring rapid profiling of large sample sets or dynamic studies relevant to industrial applications. Proton transfer reaction time‐of‐flight mass spectrometry (PTR‐ToF‐MS) is particularly well suited for tracking volatile release in real time. This technique combines soft ionization with direct, solvent‐free headspace sampling, eliminating the need for preconcentration or chromatography and largely preserving protonated molecular ions while minimizing fragmentation [26]. By providing rapid, real‐time measurements, it enables monitoring of melting dynamics through the continuous tracking of headspace volatiles at different temperatures and states (i.e., from frozen to melted). Therefore, this technique can help to characterize potential differences in VOCs released by different hydrocolloids in a complex food system, such as ice cream.

To the authors' knowledge, this study marks the first application of PTR‐ToF‐MS to investigate aroma release in ice cream as a function of stabilizer blends. By comparing stabilizers used as single ingredients, binary and ternary blends, and a conventional industry standard, we tracked VOCs profiles across both the frozen and melted states of the ice cream. A peach aroma formulation was selected as an ideal model system for this purpose, as its key constituent compounds, isoamyl acetate, cis‐3‐hexenyl acetate, linalool, and furaneol, exhibit a wide range of volatility, polarity, and hydrophobicity; their differences in release dynamics provide an opportunity to investigate how hydrocolloid networks selectively modulate the release of esters, terpenoids, and furanones during melting.

## 2 Materials and Methods

### 2.1 Standard Solutions

Pure compounds (≥ 99% purity, Sigma‐Aldrich) of 4‐hydroxy‐2,5‐dimethylfuran‐3‐(2*H*)‐one, linalool, cis‐3‐hexenyl acetate, and isoamyl acetate were purchased from Sigma‐Aldrich Trading Co. Ltd. (Milan, Italy) to identify the fragmentation patterns of each compound during the PTR‐ToF‐MS headspace analysis. Stock solutions (100 ppm) of each aroma compound were first prepared in ethanol (≥ 99%, Sigma‐Aldrich). Working solutions of 1 and 50 ppm were obtained by diluting the stock solution in distilled water, except 4‐hydroxy‐2,5‐dimethylfuran‐3‐(2*H*)‐one, whose stock solution was prepared in Milli‐Q water.

### 2.2 Ice Cream Formulation

Twelve different ice cream formulations were produced by adding 63.4% demineralized water, 11% skimmed milk powder, 11% sugar, 7.5% coconut oil, 4.5% glucose syrup 39DE, 2% sunflower oil, 0.32% emulsifiers, and 0.28% stabilizers. Detailed information on stabilizer blends is shown in Table 1. For aroma‐containing formulations, a peach aroma mixture was incorporated at 0.5% (w/w), resulting in final concentrations of 0.0045% 4‐hydroxy‐2,5‐dimethylfuran‐3‐(2*H*)‐one, 0.0025% isoamyl acetate, 0.0020% linalool, and 0.0015% cis‐3‐hexenyl acetate. All the ingredients were obtained from the industrial partner (Soremartec S.r.l., Alba, Italy).

**TABLE 1:** Detailed ice cream formulatio, ingredient percentages and stabilizer blend composition.

Ingredient	Amount (%)	Blend of stabilizers	Composition (%)
Stabilizers	0.28	Control (NS)	—
		Alginate (A)	0.28%
		Guar (G)	0.28%
		Locust bean (L)	0.28%
		Xanthan (X)	0.28%
		Alginate + guar (AG)	0.14% each
		Alginate + xanthan (AX)	0.14% each
		Guar + xanthan (GX)	0.14% each
		(A) + (G) + kappa carrageenan (K)	0.13% A + 0.13% G + 0.01% K
		(A) + (L) + kappa carrageenan (K)	0.13% A + 0.13% L + 0.01% K
		(A) + (X) + kappa carrageenan (K)	0.13% A + 0.13% X + 0.01% K
		(L) + (G) + kappa carrageenan (K)	0.13% L + 0.13% G + 0.01% K

Different formulations were produced by only varying stabilizer combinations. These included a control sample without stabilizers with peach aroma (NSF) and without peach aroma (NS), along with samples containing single, binary, andternary mixes, each mix being both aroma‐containing (_F) and without aroma (_N). The stabilizers evaluated were guar gum, locust bean gum, xanthan gum, alginate, and kappa carrageenan. The formulations were designed to evaluate (i) single‐gum systems, to isolate individual stabilizer effects; (ii) selected binary blends, to emphasize synergy with alginate, a more innovative ingredient for ice cream; and (iii) ternary blends resembling a commercial ice cream stabilizer system.

### 2.3 Ice Cream Preparation

The ice cream mixes were prepared by manually blending the dry ingredients (sugar and stabilizers) thoroughly to ensure uniform dispersion [1]. This dry mix was slowly added to the milk base with continuous stirring on a cooktop over medium heat. The remaining ingredients were added at temperatures appropriate for their solubility, ranging from 20°C to 50°C. The mixture was then pasteurized at 80°C for 30 s and homogenized using an Ultra‐Turax homogenizer (IKA, Staufen, Germany) operating at maximum speed (25 000 rpm) for 30 s. After homogenization, the mix was cooled to 4°C on an ice bath and aged in the fridge (4°C) overnight to allow stabilizer hydration and mix stabilization. For aroma‐containing samples, the peach aroma compounds (Table 2) were added to the aged mix at a concentration of 0.5% (w/w), with gentle stirring to ensure even distribution. The ice cream mix was frozen and aerated in a batch freezer (Domo DO9O66I, Belgium) at −5°C for 20 min; the final temperature of −25°C was held for the final 10 min. The semisolid ice cream was packed into 400‐mL airtight containers and hardened at −20°C for 7 days to achieve the final ice cream structure.

**TABLE 2:** Physicochemical properties of the peach aroma compound. Measured ion masses and relative intensities (values in parentheses are normalized percentages [27]).

Molecule	Formula	LogP	Vapor pressure (Pa at 25°C)	BP (°C)	ODT (μg kg^−1^)	MW (g mol^−1^)	Protonated ions (*m/z*)	Fragments (*m/z*)
Cis‐3‐hexenyl acetate	C_8_H_14_O_2_	2.83	162.5	75	7.8^a^	142.2	143.107(21)	101.096(1), 83.086(82), 61.017(5), 43.018(100)
Isoamyl acetate	C_7_H_14_O_2_	2.25	746.6	142	1.6^b^	130.18	131.107(66)	71.086(100), 61.017(11), 43.018(51)
Linalool	C_10_H_18_O	2.97	12.1	198	4^a^	154.25	155.143(0.04)	137.133(100), 121.065(0.01)
4‐Hydroxy‐2,5‐dimethyl‐3‐(2*H*)‐furanone	$C_{6}H_{8}O_{3}$	0.95	4.3	216	160^c^	128.13	129.055(78)	111.044(100)
γ‐Undecalactone	$C_{11}H_{20}O_{2}$	3.3	0.4	297	7.2^d^	184.28	ND	ND
γ‐Decalactone	C_10_H_18_O_2_	2.7	1.2	266	24.8^d^	170.25	ND	ND
δ‐Decalactone	$C_{10}H_{18}O_{2}$	2.5	1.1	281	36.8^d^	170.25	ND	ND

*Note:* LogP and molecular weight (MW) values were retrieved from the PubChem database. Boiling point (BP) values at 760.00 mmHg and vapor pressure at mmHg were obtained from the database of The Good Scents Company, a factor of 133.3223684 was used to convert mmHg to Pa. Odor threshold (ODT) values were obtained from literature: a,b,c,d [28, 29, 30]. ND stands for not detected.

### 2.4 PTR‐ToF‐MS Analysis

#### 2.4.1 Sample Preparation

For the ice cream samples prepared in triplicates, 1.75 ± 0.25 g of each formulation (both aroma‐containing and without aroma) were weighed into 20 mL GC vials and stored overnight at –20°C, empty vials followed the same storage treatment and were used as blanks. Headspace measurements were performed sequentially at two distinct points: first, immediately after extraction from the cold storage to capture the frozen state, and second, following a 20‐min incubation at 30°C to capture the melted state.

#### 2.4.2 Sample Measurement

VOCs were measured using a proton transfer reaction time‐of‐flight mass spectrometer (PTR‐ToF‐MS 8000; IONICON Analytik, Innsbruck, Austria) coupled to an adapted multipurpose autosampler (MPS Multipurpose Sampler, GERSTEL, Mülheim an der Ruhr, Germany) and a static headspace module (IONICON Analytik, Innsbruck, Austria). The instrument was operated at a drift tube temperature of 110°C, with a drift voltage of 620 V, and a drift pressure of 2.8 mbar, resulting in an E/N ratio of 128 Townsend (Td), with E corresponding to the electric field strength and N to the gas number density (1 Td = 10^−17^ Vcm^2^). A small aliquot of the headspace was transferred through a heated inlet (110°C) at a flow rate of 90 sccm.

Sample vials were placed in an autosampler equipped with temperature‐controlled coolers. Standards (1.5 mL in GC vials) were equilibrated at 20°C for 20 min, whereas ice cream samples underwent two sequential equilibrations, first at 4°C for 1 min (capturing the frozen ice cream headspace), then after 20 min at 30°C (capturing the melted state). To minimize carryover, the 2.5‐mL syringe was flushed with 150 sccm of nitrogen (N_2_) for 30 s prior to each headspace withdrawal. Then, 1.5 mL of equilibrated headspace was withdrawn at a speed of 500 μL s^−1^. The withdrawn headspace was injected into the headspace (HS) module over ~25 s at 100 μL s^−1^, resulting in a ~16‐fold dilution. During SHS analysis, zero air flowed at 90 sccm to maintain stable conditions. Mass spectra were acquired at 1 spectrum per second, providing a mass resolution of at least 4000 M/ΔM, sensitivity (> 10 cps ppbV^−1^), and LOD (< 100 pptv at 1 spectrum per second) [31].

#### 2.4.3 Data Extraction and Processing

PTR‐ToF‐MS data were processed using in‐house software developed in MATLAB (MathWorks, Natick, Massachusetts, USA), using the procedure described elsewhere [32] for internal calibration, noise reduction, baseline correction, and concentration calculations. Absolute headspace VOC concentrations (ppbV) were calculated from the mass peak intensities according to the equation by Lindinger et al., assuming a constant reaction rate coefficient of 2 × 10^−9^ cm^3^ s^−1^. This approach may introduce a systematic error of less than 30% for each compound's estimated concentration, yet it provides a robust estimate of VOC levels for comparative purposes [33]. The data extraction of PTR‐ToF‐MS spectra resulted in 240 mass peaks for H_3_O^+^. From these, only *m/z* signals showing intensities significantly higher than the blanks (*p* < 0.01) were retained [34]. Additionally, aroma‐containing and no‐aroma ice cream were compared with identify *m/z* signals attributed to added peach aroma; a total of 11 *m/z* signals molecules were kept for further statistical analysis. Pure standards of each target compound were analyzed using the same headspace preparation protocol as the ice cream samples to validate the selected mass peaks.

#### 2.4.4 Data Analysis

Univariate statistical analyses were performed to the four target peach compounds. For each compound, the summed intensities of the protonated molecular ion and the major fragment ion was used to quantify the signal: isoamyl acetate (*m/z* 131.107 + 71.086), linalool (*m/z* 155.143 + 137.133), cis‐3‐hexenyl acetate (*m/z* 143.107 + 83.086), and furaneol (*m/z* 129.055 + 111.044). For each compound, the summed signal (ppbV) obtained from three replicates per sample was analyzed by one‐way ANOVA with *Sample* as a fixed factor. When ANOVA was significant (*p* < 0.05), pairwise differences among samples were evaluated using Tukey's HSD post hoc test, and compact‐letter groupings were added to barplots.

A principal component analysis (PCA) was performed on the autoscaled intensities of the 11 selected mass peaks (*m/z* 43.018, 61.017, 71.086, 83.086, 101.069, 111.044, 129.055, 131.107, 137.133, 143.107, 155.143) at 1 (4°C) and 20 min (30°C). The resulting scores and loadings were visualized as a biplot with group ellipses representing sample groupings according to sampling time (1 vs. 20 min) and aroma condition (with vs. without added aroma). Because PCA was intended to capture the overall multivariate aroma pattern rather than test single‐compound differences, the set of *m/z* variables used for PCA was broader than that used for ANOVA. In this sense, the statistical strategy includes the identification and sum of (i) protonated molecular ions of the peach aroma compounds and (ii) major fragments based on the injection profile of the pure standard solutions. This was done to increase signal intensity and have a better estimation of the total release for each compound.

Odor activity values (OAVs) were calculated for the key peach aroma compounds: hexenyl acetate, isoamyl acetate, linalool, and furaneol. The calculation was based on the formula OAV=C/OT [35], where each compound concentration (*C*) is divided by its orthonasal detection threshold (*OT*), using theoretical threshold values reported in aqueous media (Table 2). The OAVs of each key peach aroma compound were summed to obtain a total OAV for each sample. On these values, one‐way ANOVA, followed by Tukey's HSD post hoc test for pairwise comparisons, with results shown as barplots ± SD and letter annotations.

All statistical analyses were carried out in R (version 4.0.2) with packages such as *tidyverse*, *emmeans*, *multcompView*, *factoextra*, and *ggplot2*.

## 3 Results and Discussion

### 3.1 Mass Selection and Fragment Identification

PTR‐ToF‐MS is known for its soft ionization, which minimizes analyte fragmentation compared with electron impact techniques, thereby simplifying mass spectra and improving identification. However, fragmentation can still occur, especially for compounds like alcohols, aldehydes, and esters [36]. For this reason, the initial phase of this study focused on identifying and characterizing VOCs within the peach aroma by analyzing pure standard compounds. The target mass peaks and their identified fragments are shown in Table 2, together with their physicochemical properties. These properties are particularly relevant for interpreting differences in aroma release observed later in the study. In particular, physicochemical properties (e.g., vapor pressure, boiling point [Bp], molecular weight [Mw], and *n*‐octanol–water partition coefficient [LogP value]). Together, these molecular features govern diffusion within the matrix and determine the compound's gas/solution partitioning.

For each compound, the expected *m/z* ratio of the protonated molecule [M + H]^+^ and its main fragment ions obtained from the most common fragmentation pathways under proton transfer conditions were reviewed via literature and online databases [27, 36, 37, 38]. For esters, the primary fragmentation pathway involves cleavage of the alkyl–oxygen bond in the ester group [R–O–C(=O)–CH_3_] along with the formation of characteristic acylium cations (e.g., CH_3_CO^+^, *m/z* 43) [39]. These fragmentation hypotheses were validated by performing PTR‐ToF‐MS measurements on standard solutions of each compound. Table 2 shows that isoamyl acetate produced a dominant protonated molecular ion at *m/z* 131.107 together with a characteristic fragment at *m/z* 71.086, resulting from cleavage of the ester bond. Similarly, cis‐3‐hexenyl acetate exhibited a protonated molecular ion at *m/z* 143.107 and a fragment ion at *m/z* 83 corresponding to the loss of acetate, consistent with previous PTR‐ToF‐MS studies of ester compounds [28]. Linalool exhibited a protonated molecular ion at *m/z* 155.143 and the fragment at *m/z* 137, probably due to dehydration [27]. For furaneol, the principal fragment was observed at *m/z* 111.044, suggesting dehydration or neutral loss reactions from the protonated molecule [40]. The combination of protonated molecular ions and their characteristic fragments provided a reliable basis for compound identification and for interpreting stabilizer aroma retention results.

**FIGURE 1:**
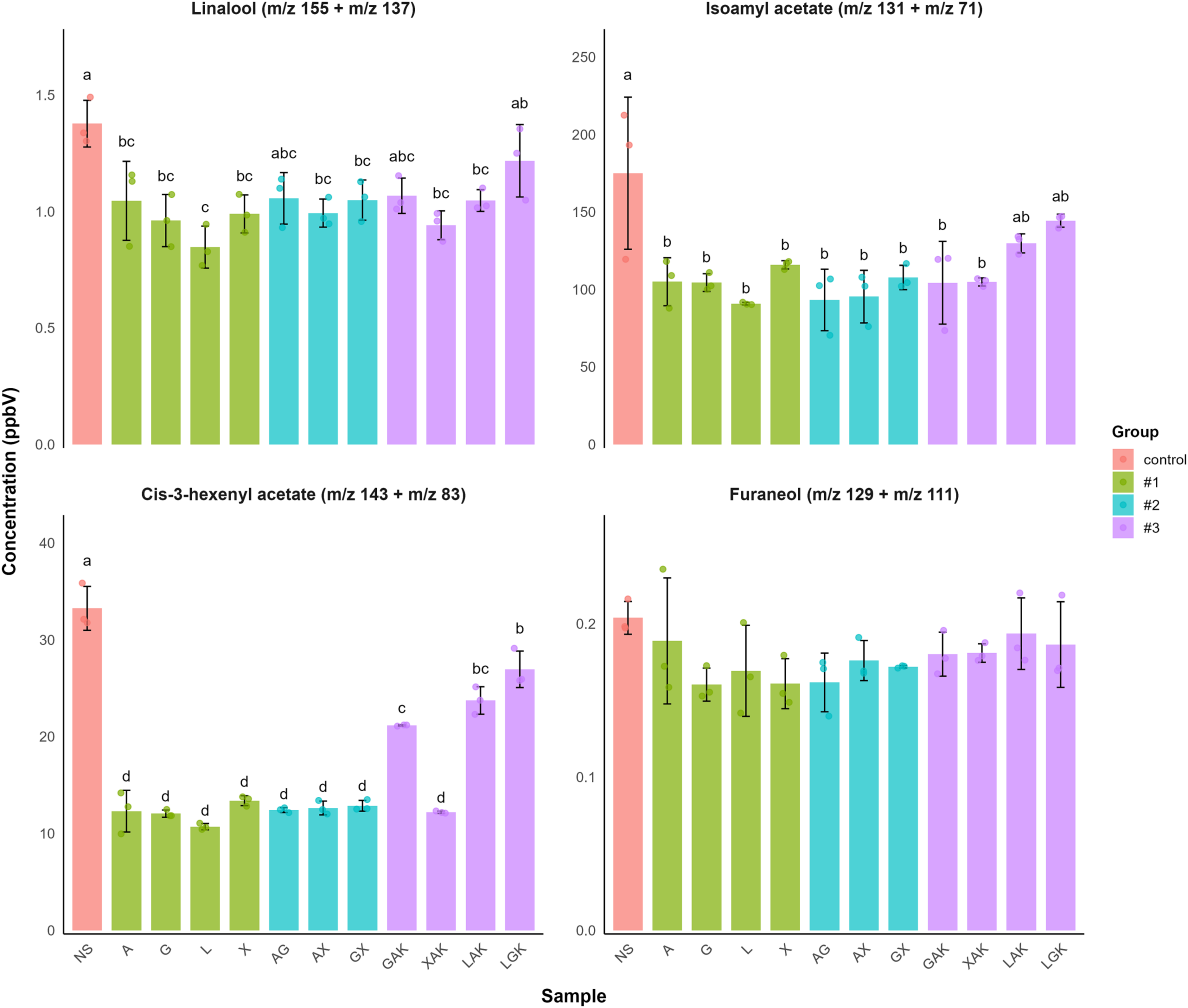
Release of target VOCs from the melted ice cream matrix: Linalool (*m/z* 155.143 and its fragment 137.133), isoamyl acetate (*m/z* 131.107 and its fragment 71.086), cis‐3‐hexenyl acetate (*m/z* 143.107 and its fragment 83.086), and furaneol (*m/z* 129.055 and its fragment 111.044). Samples are colored by group: red, control (no stabilizer); olive green (one stabilizer); blue (two stabilizers); and purple (three stabilizers). Data were analyzed using one‐way ANOVA. Significant effects of formulation were observed for linalool, isoamyl acetate, and cis‐3‐hexenyl acetate (*p* < 0.001), whereas furaneol did not show significant differences (*p* = 0.27). Different letters above the bars indicate significant differences between formulations (Tukey's HSD post hoc test, *p* < 0.05). Panels without letter designations (furaneol) indicate the absence of statistically significant differences across samples.

The aroma compounds investigated in this study were chosen to reproduce a commercially relevant peach flavor used in industrial ice cream and exhibited a range of physicochemical properties (Table 2). Because ice cream contains a substantial fat fraction, compounds with higher LogP values are expected to partition preferentially into the fat phase, reducing their concentration in the aqueous phase and limiting their release into the headspace. For example, cis‐3‐hexenyl acetate shows a higher hydrophobicity than furaneol (LogP = 2.8 vs. 0.95) and therefore furaneol should likely be more abundant in the gas phase. However, a higher concentration of cis‐3‐hexenyl acetate was measured, due to its lower boiling point and higher vapor pressure when compared with furaneol (BP = 75°C, LogP = 2.8, VP = 162.5 Pa vs. BP = 216°C, LogP = 0.95, VP = 4.3). These parameters are useful for interpreting the PTR‐ToF‐MS signals and the differences in volatile release observed in the subsequent results (Figure 1).

Linalool and isoamyl acetate exhibit relatively similar hydrophobicity and molecular weight (LogP = 2.97 and 2.25; MW = 154.25 and 130.18 g mol^−1^) but differ in volatility‐related properties. Isoamyl acetate has a lower boiling point and higher vapor pressure (BP = 142°C and VP = 746.6 Pa) than linalool (BP = 198°C and VP = 12.1 Pa). These physicochemical differences influence the balance between retention within the ice cream matrix and transfer to the gas phase. The higher vapor pressure of isoamyl acetate increases its tendency to partition into the headspace, resulting in a greater measured concentration despite its comparable hydrophobicity to linalool. A previous study by Savary et al., investigated aroma retention in acacia gum solutions by measuring gas–liquid partition coefficients using HS‐GC with a flame ionization detector and the phase ratio variation method, showed that lower boiling point, smaller, and more mobile molecules readily populate the headspace in pure aqueous solution, as observed with isoamyl acetate (Figure 1).

Heavier and less volatile VOCs are less readily released in the headspace and detected by PTR‐ToF‐MS [26]. This reason could explain why the lactones part of the peach aroma mix (δ‐decalactone, γ‐decalactone, and γ‐undecalactone) were not detected in either the ice cream headspace or standard solutions (Table 2) and therefore not considered. In addition, these lactones are more hydrophobic (LogP = 2.5–3.3) than isoamyl acetate (LogP = 2.25), which may further promote their partitioning into the fat phase and reduce their release into the headspace. Consequently, only a fraction partitions into the gas phase, often producing signals close to or below the detection limit of PTR‐ToF‐MS. Furthermore, medium‐mass lactones experience adsorption losses on inlet lines. These analytical challenges have been previously reported, particularly for relatively low‐volatility compounds in the 150–200 *m/z* range and for molecules prone to fragmentation or surface losses during sampling and ionization [41]. For this reason, lactones are frequently analyzed using preconcentration techniques such as HS‐SPME‐GC‐MS. This approach enhances extraction efficiency from the food matrix and concentrates the volatile analytes prior to chromatographic separation, thereby enabling the detection of trace‐level compounds in the headspace [42].

### 3.2 Effects of Stabilizers

The interplay between hydrocolloids and VOCs is important for understanding differences in aroma perception of complex food matrices such as ice cream. To elucidate these interactions, headspace concentrations (ppbV) of key peach aroma compounds were monitored across various ice creams that varied in the stabilizer blend present.

The measurements obtained in the frozen state (1 min at 4°C) were intentionally excluded from this compound‐by‐compound comparison because in the frozen state, the ice crystal network and viscous unfrozen phase trap and restrict volatile diffusion, yielding uniformily low headspace concentration across all formulations. Upon phase transition, the results obtained from the melted ice cream are shown in Figure 1. The retention of peach aroma volatiles differed significantly among hydrocolloid blends across three out of the four target ions: isoamyl acetate (*m/z* 131.107 + 71.086), linalool (*m/z* 155.143 + 137.133), cis‐3‐hexenyl acetate (*m/z* 143.107 + 83.086), except for furaneol (*m/z* 129.055 + 111.044). The control (NS) exhibited the highest volatile release, particularly for isoamyl acetate, indicating minimal retention in the absence of hydrocolloids.

Among the individual stabilizers, L showed the strongest retention, with significantly lower release (*p* value < 0.05) compared with blends like LGK, LAK, GAK, and NS for cis‐3‐hexenyl acetate; L was also significantly lower than LGK and NS for linalool. The magnitude of the stabilizer effect varied among aroma compounds; cis‐3‐hexenyl acetate showed the largest differences between formulations, particularly for the LGK blend. In contrast, furaneol release was not significantly affected by stabilizer compositions (*p* value = 0.27), and no significant pairwise differences among formulations were detected. Stabilizer‐induced retention is not uniform across all aroma compounds, with more volatile esters exhibiting greater sensitivity to matrix modifications than furaneol under the evaluated conditions.

The differences in aroma release observed in Figure 1 can also be related to the physicochemical properties of the analyzed molecules (Table 2). The selected aroma compounds exhibited distinct hydrophobicity and volatility, which influenced their partitioning between the matrix and the headspace. For example, cis‐3‐hexenyl acetate and isoamyl acetate have moderate LogP values (2.83 and 2.25) and relatively low boiling points (75°C and 142°C), which favor their transfer into the headspace. In contrast, furaneol has a much lower LogP value (0.95) and a higher boiling point (216°C), indicating higher polarity but lower volatility; these characteristics likely contributed to the absence of significant differences in furaneol release among the samples.

Aroma release in ice cream is influenced by the partitioning of VOCs among the fat, aqueous, and gas phases, as well as by interactions with stabilizers. Hydrocolloids, as hydrophilic polysaccharide macromolecules, modulate aroma release through two mechanisms: increasing the serum‐phase viscosity, which inversely relates to aroma diffusion, and direct interactions with aroma molecules via adsorption, entrapment, encapsulation, and hydrogen bonds [43]. Conversely, more hydrophilic compounds with lower LogP < 3 values, such as certain aldehydes, diffuse out more readily. Our data for isoamyl acetate (LogP = 2.25) are consistent with this behavior, showing relatively low retention. Importantly, such differential retention not only modifies the overall intensity of aroma release but can also shift the qualitative perception of the flavor profile, because aroma perception arises from the balance of multiple volatiles with distinct odor notes. In this way, hydrocolloids may indirectly change not just how strong a peach aroma is perceived, but also which descriptors (e.g., fruity, floral, and green) dominate the sensory experience.

For most single‐, binary‐, and ternary‐stabilizer blends, the release of most aroma molecules is similar in concentration, apart from the blend LGK. Previous research has shown that under static headspace, aroma release is dominated by molecular diffusion, which is inversely related to the continuous‐phase viscosity [13]. Another study focused on simple models has justified retention based on polysaccharide‐aroma binding, investigating galactomannan structure where variations in their M/G ratios of locust bean gum and guar gum will lead to influences on volatile retention, mainly due to enhanced hydrophobic intramolecular and intermolecular clustering around their galactose‐rich regions [44]. However, no significant differences in aroma release were observed between the individual locust bean gum and guar gum formulations in the present study. Therefore, the observed retention patterns cannot be attributed solely to differences in galactomannan structure and are likely influenced by the broader matrix composition and interactions among hydrocolloids when complex foods are investigated.

The synergistic network effect in the triple‐gum blends combines (i) electrostatic gelation via k‐carrageenan anionic sulfates crosslinked to k‐casein, (ii) the viscosity generated by galactomannans, and (iii) hydrophobic binding sites. This matrix, characterized by a more ordered, porous structure, affords both diffusion and aroma entrapment, yielding the observed results on intermediate release for LGK and LAK. The common use of the blend containing locust + guar and kappa carrageenan for commercial formulations reflects its ability to form a product with high aroma release.

Overall, these results confirm that hydrocolloids alter volatile release in a compound‐dependent manner, driven by differences in aroma compound hydrophobicity and the specific interactions between each aroma compound and the complex polymer matrix formed by the hydrocolloids. Moreover, the mechanical character of the polysaccharide network in the unfrozen phase (rigidity vs. flexibility) directly influences ice cream texture: flexible polysaccharides (e.g., locust bean) form softer, more flavorful ice cream, whereas rigid gums (e.g., xanthan) yield firmer and less flavorful ice creams [45].

### 3.3 Overview of Aroma Release Profiles

Differences in the release patterns of individual aroma compounds across stabilizer formulations likely result from the combined effects of stabilizer network properties (viscosity, binding sites, and gel structure) and the physicochemical characteristics of the aroma molecules. To capture these overall variations, PCA was performed to explore the volatile profiles of different ice creams both in the frozen and melted states, as well as to discriminate between flavored and unflavored formulations.

**FIGURE 2:**
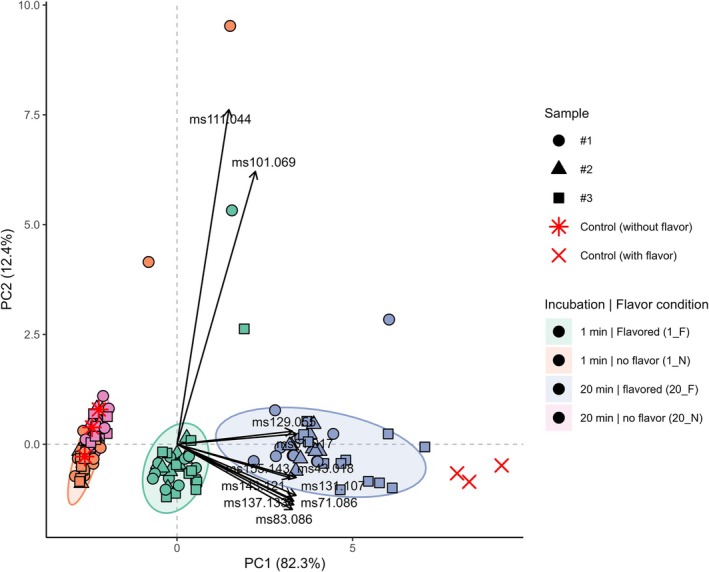
PCA score plot of autoscaled PTR‐MS data showing individual replicates to obtain within‐sample variability. Colors indicate the sampling time and flavor condition: 1_F (green), 1_N (orange), 20_F (blue), and 20_N (pink), where F denotes flavored and N denotes nonflavored formulations. Symbol shapes distinguish between the three stabilizer groups (1, circles; 2, triangles; 3, squares). The flavored control (NSF) is represented by a red cross (X), whereas the nonflavored control is represented by a red asterisk (*), both measured at 20 min. Ellipses represent the dispersion of samples within each time (1 or 20 min)/flavored (_F or _N). Three replicates are shown for each stabilizer blend.

PCA was performed on the autoscaled data from the two sampling points (1 min at 4°C and 20 min at 30°C) across aroma‐containing (_F) and aroma‐free (_N) ice cream samples (Figure 2). The analysis was conducted on the individual replicate measurements (*n* = 3 per stabilizer group); *m/z* considered were 43.018, 61.017, 71.086, 83.086, 101.069, 111.044, 129.055, 131.107, 137.133, 143.107, 155.143. The first two principal components explained a large portion of the total variance of 94.7%, revealing four distinct ellipses. Along PC1, the peach aroma‐containing samples are distributed predominantly on the positive side, whereas the aroma‐free ice creams cluster on the negative side. PC2 showed some minor subgrouping likely reflecting differences in stabilizer type.

The tight clustering and minimal overlap among these groups underscore that both the recipe (aroma vs. aroma‐free) and the time‐temperature conditions (1 min at 4°C vs. 20 min at 30°C) impart clear, measurable differences in the VOCs profile. In contrast, the aroma‐free samples at 1 (1_N) and 20 min (20_N) nearly coincide because, without added peach esters and alcohols, their headspace is lacks peach‐derived volatiles.

Our results demonstrate that temperature and matrix composition together govern volatile release in ice cream, and PTR‐ToF‐MS is key to unraveling these dynamics in real time. In the frozen state, aroma release is expected to be strongly suppressed as a rigid ice network and high serum viscosity inhibit aroma diffusion; PTR‐ToF‐MS captures this suppression quantitatively by showing low headspace signals in the frozen state. As melting progresses, ice crystals melt, and the serum‐phase viscosity reduces. Simultaneously, the increase in temperature enhances the aroma compounds' partitioning into the headspace, tracked by PTR‐ToF‐MS, which records the burst of key *m/z* signals at 20 min. Furthermore, hydrocolloids and other stabilizers form three‐dimensional networks that can bind or entrap aroma molecules [13]. Due to the multiphase system of ice cream, the mechanism underlying aroma retention cannot be fully isolated. Further studies using simplified serum‐phase model systems could help clarify the relative contributions of stabilizers to the aroma–matrix interactions.

**FIGURE 3:**
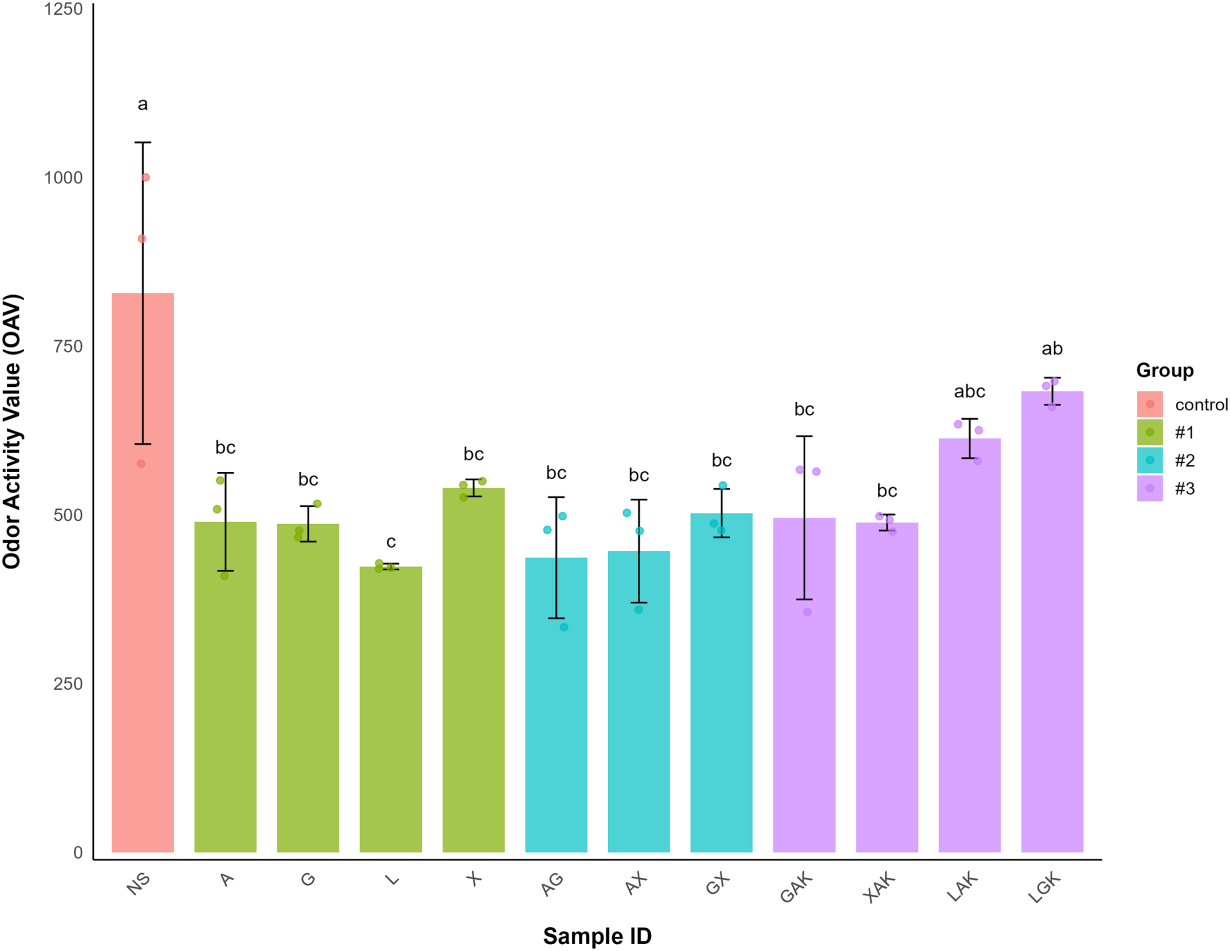
Total odor activity values (OAVs) across different ice cream formulations varying in stabilizer blend. Bars represent the mean OAVs calculated as the sum of individual OAVs for cis‐3‐hexenyl acetate, isoamyl acetate, linalool, and furaneol, with error bars indicating the standard deviation. Different letters indicate statistically significant differences, one‐way ANOVA (*p* < 0.05) followed by Tukey's HSD post hoc pairwise comparisons.

The real‐time capabilities of PTR‐ToF‐MS's enable in vitro monitoring of these volatile dynamics within the complex ice cream matrix, validating both suppression in the frozen state and rapid release upon melting. While in vitro instrumental measurements of VOCs release are fundamental, their direct impact on sensory perception is rather complex, given the influence of numerous individual physiological and psychological factors [46]. To better estimate the potential sensory impact assessment of the total peach aroma (Figure 3), OAV were calculated and summed based on the key aroma compounds of the melted sample headspace: cis‐3‐hexenyl acetate (*m/z* 143.107 + 83.086), isoamyl acetate (*m/z* 131.107 + 71.086), linalool (*m/z* 155.143 + 137.133), and furaneol (*m/z* 129.055 + 111.044).

Interestingly, while the LGK and LAK samples showed comparatively higher increases in the *m/z* during the analysis of isoamyl acetate and cis‐3‐hexenyl acetate (Figure 1), their OAVs in the melted state (Figure 3) followed the same trend, ranking among the highest stabilizer blends; their values remained generally lower than the nonstabilized control (NS). This suggests that LGK and LAK may be particularly relevant blends, where certain stabilizer combinations may better preserve aroma availability despite the generally suppressive effects of the stabilizers on volatile release. These results highlight the value of considering both in vitro aroma release and OAV predictions, and they underscore the importance of validating instrumental findings against dynamic sensory evaluation to fully understand aroma delivery in ice cream.

Such partial agreement between volatile release and OAV predictions mirrors observations in other complex food matrices, where instrumental measurements capture trends but do not fully explain sensory outcomes. For example, Arancibia et al. [47] showed that thickeners and fat content in dairy desserts significantly altered both in vivo release and perceived intensity, with trends not always matching absolute release values. Likewise, Hort [48] reported that increased viscosity reduced perceived aroma intensity even when volatile concentrations remained unchanged, underscoring the modulatory role of texture. More broadly, Le Quéré and Schoumacker [49] highlighted that flavor perception is a dynamic process shaped by volatile release patterns, oral processing, and retro‐nasal transport, making it necessary to pair PTR‐ToF‐MS with in vivo analysis (nose space) with sensory time‐intensity methods.

Future work should focus on how textural differences impact aroma diffusion and perception. First, employ simplified model systems to isolate the effect of hydrocolloids on aroma release. Building on this, integrate time‐intensity sensory testing (e.g., nose‐space PTR‐ToF‐MS coupled with dynamic sensory evaluation) to map how PTR‐ToF‐MS signals and OAV correspond to the temporal evolution of aroma perception during consumption. Finally, because literature odor thresholds often derive from aqueous systems, determining threshold values within the milk matrix will refine OAV calculations and improve sensory predictions for complex milk‐based foods.

## 4 Conclusion

This study demonstrates that both temperature‐driven melting and hydrocolloid composition are critical factors governing the release of aroma volatiles from ice cream. By using rapid, noninvasive PTR‐ToF‐MS on frozen samples, we quantified how aroma signals are suppressed under a rigid ice network and the rapid release of VOCs as melting opens diffusion pathways. The results further demonstrate that stabilizer composition modulated aroma release in a compound‐specific manner. Specifically, the LGK and LAK blends exhibited higher volatile release than other systems, suggesting that the matrix structures formed by these hydrocolloid combinations were less effective at retaining aroma compounds when melted. Although the underlying mechanism was not directly investigated, differences in serum‐phase organization, volatile partitioning, and hydrocolloid–aroma interactions may have contributed to the observed release patterns.

The OAV provided an estimate of the potential sensory impact, showing that, in terms of aroma perception, there should be significant changes by varying the hydrocolloid. These observations were consistent with the instrumental in vitro results, which showed enhanced release of specific VOCs in formulations such as LGK and LAK. Overall, the findings highlight an important effect of stabilizers in modulating aroma delivery during melting. These findings also highlight the need for complementary sensory evaluation to better link instrumental VOCs release with aroma perception. In addition, the ODT values used for OAV calculations are often derived from aqueous systems, and when analyzing complex food systems, determining the odor threshold values directly in milk‐based matrices could improve the accuracy of sensory predictions in ice cream.

## Funding

This work is financially supported by the Italian Ministry of University and Research (MUR) under the National Recovery and Resilience Plan (PNRR) funded by the European Union NextGenerationEU with industrial cofunding from Soremartec Ferrero Group.

## Data Availability

The data that support the findings of this study are available from the corresponding author upon reasonable request.
